# Multistate Outbreaks of Foodborne Illness in the United States Associated With Fresh Produce From 2010 to 2017

**DOI:** 10.3389/fmicb.2019.02667

**Published:** 2019-11-22

**Authors:** Christina K. Carstens, Joelle K. Salazar, Charles Darkoh

**Affiliations:** ^1^Department of Epidemiology, Human Genetics and Environmental Sciences, Center for Infectious Diseases, School of Public Health, University of Texas Health Science Center, Houston, TX, United States; ^2^Division of Food Processing Science and Technology, U.S. Food and Drug Administration, Bedford Park, IL, United States; ^3^Microbiology and Infectious Diseases Program, University of Texas MD Anderson Cancer Center UTHealth Graduate School of Biomedical Sciences, Houston, TX, United States

**Keywords:** multistate, foodborne outbreaks, produce, *E. coli*, *L. monocytogenes*, *S. enterica*, diarrheal disease

## Abstract

In the United States, the consumption of fresh fruits and vegetables has increased during recent years as consumers seek to make healthier lifestyle choices. However, the number of outbreaks associated with fresh produce that involve cases in more than one state (multistate) has increased concomitantly. As the distance along the farm-to-fork continuum has lengthened over time, there are also more opportunities for fresh produce contamination with bacterial pathogens before it reaches the consumer. This review provides an overview of the three bacterial pathogens (i.e., pathogenic *Escherichia coli, Listeria monocytogenes, and Salmonella enterica*) associated with multistate fresh produce outbreaks that occurred between 2010 and 2017 in the U.S. Possible routes of fresh produce contamination, including pre- and post-harvest, are summarized and outcomes of selected outbreaks within this timeframe are highlighted. Eighty-five multistate outbreaks linked to fresh produce with a confirmed etiology occurred from 2010 to 2017. Cross-contamination within the distribution chain and poor agricultural practices, along with the production of sprouts and importation of fresh produce were frequently implicated contributors to these events. The evolution of the food supply chain in the U.S. necessitates an examination of multistate outbreaks to shed light on factors that increase the scale of these events.

## Introduction

The consumption of fresh fruits and vegetables (“fresh produce”) has increased substantially since the 1980s due to consumer demand for a healthier lifestyle and more nutritious foods, especially in high-income countries. The World Health Organization recommends a daily intake of 400 grams of fresh produce (WHO, 2003). A diet rich in fresh produce has been shown to prevent certain chronic diseases such as diabetes, cancer, cardiovascular disease, hypertension, and obesity (WHO, 2003). Due to demand, globalization of trade has aided the distribution of fresh produce worldwide, surmounting the seasonality and location of certain commodities. In 2001, fresh produce encompassed 16.9% ($70 billion) of world agricultural trade, up from 10.6% ($3.4 billion) in 1961 (Huang, 2004). In the U.S., the *per capita* consumption of fresh produce in 2010 and 2016 was 67.2 and 68.7 kg, respectively (USDA, 2018). Although consumption of fresh produce in the U.S. is steadily increasing, only approximately 6–8% of people achieve the 400 gram daily target (Rekhy and McConchie, 2014); therefore, government and non-government agencies continuously promote nutrition education and encourage consumption of fresh produce to promote public health.

As consumption of fresh produce increases, the likelihood of associated illnesses and outbreaks caused by microbial pathogens is also expected to increase. Since fresh produce is often consumed in its raw state with no processing step to eliminate harmful organisms, there is the potential for contamination with foodborne pathogens and thus, illness upon consumption. For instance, 1779 foodborne outbreaks with a confirmed food vehicle and a confirmed etiology occurred in the U.S. from 2004 to 2010, of which 9.2% (163) were attributed to fresh produce (CDC, 2017d). Out of the 163 produce-associated outbreaks, 27.6% (45) occurred in multiple states of the U.S. A multistate outbreak is defined as an outbreak caused by the same contaminated food item that has been distributed to multiple states. Multistate produce-associated outbreaks from 2004 to 2010 caused a total of 4949 illnesses, 895 hospitalizations, and nine deaths. From 2010 to 2017, 1797 foodborne outbreaks with a confirmed food vehicle and a confirmed etiology occurred in the U.S., of which 12.7% (228) were attributed to fresh produce (CDC, 2017d). Out of these 228 produce-associated outbreaks, a total of 37.3% (85) were multistate. These multistate produce-associated outbreaks resulted in 4748 illnesses, 1190 hospitalizations, and 55 deaths. Overall, the number of produce-associated foodborne outbreaks increased in the U.S. from 2010 to 2017 as compared to 2004 to 2010. The number of produce-associated multistate outbreaks also increased, as did the number of deaths due to these outbreaks. These data suggest that multistate produce-associated outbreaks in the U.S. are on the rise, which is of critical public health concern.

The U.S. Food and Drug Administration (FDA) Food Safety Modernization Act (FSMA) was signed into law in 2011 and allows the FDA to not only respond to food contamination events and foodborne outbreaks but also to focus on prevention-based strategies (FDA, 2019a). The Produce Safety Rule, part of FSMA, provides science-based guidance for safe production, harvest, and handling of fruits and vegetables (FDA, 2019b). The final rule went into effect in January 2016 and indicates that all fresh produce facilities implement mandatory preventive controls and produce safety standards. The key requirements of the rule include a minimum quality for agricultural water, microbial standards for biological soil amendments, specifications for the use of domesticated animals for grazing or working, worker training and hygiene, and standards for tools, equipment, and buildings. The rule also provides draft guidance for sprout growers. In 2017, a total of 13 multistate produce-associated outbreaks occurred in the U.S., whereas the five preceding years (2010–2016) averaged 14 outbreaks per year. Although the number of average outbreaks has not changed largely since the enactment of the final rule, it may be too soon to detect the full effect of the new regulation.

This review will highlight the multistate produce-associated outbreaks in the U.S. from 2010–2017 and the associated bacterial pathogens: *Listeria monocytogenes*, *Escherichia coli*, and *Salmonella enterica*. Further discussion will involve various routes of fresh produce contamination, both pre- and post-harvest, and the risks associated with individual categories of fresh produce including fruits, leafy greens, and vegetable row crops. Finally, this review also explores the incidence and contributing factors of multistate foodborne outbreaks associated with fresh produce consumption. A comprehensive overview of the principal causes and outcomes of multistate outbreaks may establish patterns that can aid in the reduction and possible prevention of these events.

## Pathogens Associated With Fresh Produce

Human pathogens generally associated with fresh produce include Hepatitis A, Norovirus, *Cyclospora*, *Aeromonas* spp., *Bacillus cereus*, *Campylobacter* spp., *Clostridium botulinum*, *E. coli*, *L. monocytogenes*, *S. enterica*, *Shigella* spp., *Staphylococcus* spp., and *Yersinia enterocolitica*. As there were only three bacterial pathogens involved in multistate fresh produce outbreaks from 2010 to 2017 (see section Multistate Outbreaks of Foodborne Illness Associated With Fresh Produce), this section focuses on *E. coli*, *L. monocytogenes*, and *S. enterica*.

### Pathogenic *E. coli*

*E. coli* is a Gram-negative, rod-shaped, facultative anaerobe that is associated with the gut microbiota of humans and animals (Croxen et al., 2013). *E. coli* are coliforms and common fecal indicators as they are associated with fecal matter and are found in proximity to water, soil, and animals (van Elsas et al., 2011). Some strains of *E. coli* can proliferate at temperatures as low as 7°C and as high as 46°C; optimum temperature for this organism occurs at 37°C (ICMSF, 1996). It has been determined that *E. coli* can survive in environments containing 6% sodium chloride (NaCl) and pH values > 5.4. All *E. coli* serotypes are characterized by combinations of somatic (O), flagellar (H), and capsular (K) surface antigens, which can be detected through various molecular techniques including serology (ICMSF, 1996; Nataro and Kaper, 1998).

*E. coli* can be transmitted via a foodborne route, and there are six major *E. coli* pathotypes that are broadly distinguished by virulence factors (Croxen et al., 2013; Yang et al., 2017). Enteropathogenic *E. coli* (EPEC) commonly infects children, yet can also infect adults at infectious doses of 10^8^–10^10^ CFU. Shiga-toxin producing *E. coli*/enterohemorrhagic *E. coli* (STEC/EHEC), also referred to as Verocytotoxin-producing *E. coli* (VTEC), can cause human illness with an extremely low infectious dose (<1000 CFU). The most common serotype of STEC implicated in outbreaks of foodborne disease is *E. coli* O157:H7, which is covered in the following section (see section *E. coli* O157:H7). Infection with enteroinvasive *E. coli* (EIEC) can resemble *Shigella* infection, although a greater infectious dose is required for human illness (10^6^–10^8^ CFU vs. 10–100). Both Enteroaggregative *E. coli* (EAEC) and Enterotoxigenic *E. coli* (ETEC) are frequent causes of traveler’s diarrhea and are characterized by infectious doses of 10^10^ and 10^8^ CFU, respectively. Diffusely invasive *E. coli* (DAEC) is an emerging pathotype of *E. coli* that causes persistent diarrhea in children and is suspected to contribute to Crohn’s disease in adults (Le Bouguenec and Servin, 2006). Infection with any of these six pathotypes of *E. coli* can lead to diarrheal disease. The natural history of disease does vary, however, and is dependent on the contributing *E. coli* pathotype.

More than approximately 112,000 cases of foodborne non-O157 STEC occur each year in the U.S. with a hospitalization rate of 12.8% (Scallan et al., 2011). Between 2009 and 2015, STEC was responsible for 191 (6%) of all reported outbreaks, which remained consistent from outbreaks that occurred from 1998 to 2008 (Dewey-Mattia et al., 2018). The mean total cost of non-O157 *E. coli* infections in the U.S. is 23.9 million dollars annually (Hoffmann et al., 2012). Approximately 18,000 cases of foodborne ETEC *E. coli* occur in the U.S. per year, with an additional 12,000 cases that occur due to diarrheagenic *E. coli*, not including STEC or ETEC pathotypes (Scallan et al., 2011).

#### E. coli O157:H7

Enterohemorrhagic *E. coli* O157:H7 is of particular public health concern as it is the primary cause of hemolytic uremic syndrome (HUS) in the U.S. (Banatvala et al., 2001). Transmission generally occurs via a foodborne route, from person-to-person, the consumption of contaminated water, or animal contact (Rangel et al., 1982). Symptoms vary and can include mild gastroenteritis, bloody diarrhea, hemorrhagic colitis, HUS, and even death. An estimated 63,153 cases of foodborne Shiga-toxin producing *E. coli* O157-related cases occur annually in the U.S with a hospitalization rate of 46.2%, which is a rate similar to that of *Vibrio cholerae* (43.1%; Scallan et al., 2011). The mean total cost for *E. coli* O157:H7-related illness in the U.S. is 254.8 million dollars annually (Hoffmann et al., 2012). *E. coli* O157:H7 shed in bovine and other animal feces likely causes the contamination of fresh produce and the low infectious dose required for illness increases concern (Lim et al., 2010).

### L. monocytogenes

The pathogenic bacterium *L. monocytogenes* is a Gram-positive, rod-shaped, non-spore-forming facultative anaerobe that is ubiquitous in the environment (Farber and Peterkin, 1991). Optimum growth of *L. monocytogenes* occurs between 30 and 37°C; however, the organism is highly resilient and has been observed to proliferate between temperatures of −2 to 45°C, pH values of 4.0–9.5, and NaCl concentrations as high as 10% (Liu et al., 2005). Of notable concern is the capability of *L. monocytogenes* to survive and grow at and below the U.S. FDA recommended refrigeration temperatures of ≤4°C (FDA, 2017). *L. monocytogenes* is also able to form biofilms on various surfaces, which allows for further resistance to environmental stress, and leads to difficulty with surface disinfection and sanitation (da Silva and De Martinis, 2013).

At least 12 serotypes of *L. monocytogenes* exist and are distributed into genetic categories known as lineages. Lineage I mainly comprises serotypes 1/2b, 3b, 4b, 4d, and 4e and are more often implicated in outbreaks of listeriosis (Ragon et al., 2008; Lomonaco et al., 2015). Lineage II contains serotypes 4a, 1/2c, 3a, and 3c and mainly cause sporadic cases of listeriosis. Lineages III and IV are rare in nature, with only 3% of *L. monocytogenes* belonging to this category.

The primary route of *L. monocytogenes* infection is via the consumption of contaminated food. Listeriosis can be asymptomatic or cause febrile gastroenteritis in healthy individuals, however, cases of invasive infection can result in conditions such as septicemia, meningoencephalitis, and fetal loss. Invasive listeriosis has a mortality rate of 20–30% (Swaminathan and Gerner-Smidt, 2007). Populations susceptible to listeriosis include those that are immunosuppressed, elderly, and pregnant, along with neonates. *L. monocytogenes* has an incubation period that ranges from one to 70 days based on the clinical manifestation of the infection (Hernandez-Milian and Payeras-Cifre, 2014), and an infectious dose of approximately 10^3^ cells for an immunocompromised individual (Pouillot et al., 2015).

Approximately 1600 cases of listeriosis occur in the U.S. annually (Scallan et al., 2011). Although listeriosis has a low incidence, it has the highest estimated hospitalization rate (94%) out of 31 major foodborne pathogens, including *Vibrio vulnificus* (91.3%) and *Clostridium botulinum* (82.6%). Monetary loss due to the cost of illness associated with listeriosis in the U.S. is estimated to be 2.6 billion U.S. dollars per year (Hoffmann et al., 2012).

### S. enterica

*S. enterica* is a Gram-negative, rod-shaped, non-sporulating facultatively anaerobic organism (Fabrega and Vila, 2013). Pathogenic species of *Salmonella* are of concern to the food industry as they have been documented to grow at temperatures between 8 and 45°C, pH values from 4.0 to 9.5, and water activity values as low as 0.94 (Chlebicz and Slizewska, 2018). Animal fecal materials are known to harbor *S. enterica*, which leads to the contamination of water and crops (Wiedemann et al., 2014).

Antigenic formulas are used to classify *S. enterica*, however, strains are frequently referred to by serotype name. There are six subspecies of *S. enterica* designated *enterica* (I), *salamae* (II), *arizonae* (IIIa), *diarizonae* IIIb, *houtenae* (IV), and *indica* (IV). *S. bongori* was formerly referred to as subspecies V, but is now considered a species (Crum-Cianflone, 2008). *S. enterica* subspecies *enterica* (I) contains the majority of serotypes that are human pathogens (Fierer and Guiney, 2001).

*Salmonella* spp. infect humans via various modes, such as contact with an animal carrier and the consumption of contaminated food or water. An estimated 94% of non-typhoidal *S. enterica* infections in the U.S. are foodborne (Hoffmann et al., 2012). Salmonellosis presents typical gastroenteritis symptoms, and incubation times can range between six and 48 h (Crum-Cianflone, 2008). The infectious dose has not been established, but the disease is generally self-limiting. Invasive salmonellosis can occur, especially in at-risk individuals such as children, the immunocompromised, and the elderly. Treatment with antibiotics for salmonellosis is reserved for severe cases or cases susceptible to complications (Hohmann, 2001).

Over 1 million cases of non-typhoidal *S. enterica* occur each year in the U.S., of which an estimated 27.2% result in hospitalizations (Hoffmann et al., 2012). Between 1998 and 2008, *Salmonella* spp. caused 18% of single etiology outbreaks, along with the majority of all hospitalizations (44%) and deaths (30%) recorded. *Salmonella* spp. also caused 53% of all reported multistate outbreaks in the same time period (Gould et al., 2013). Between 2009 to 2015, *S. enterica* Enteritidis caused the largest of 177 multistate outbreaks by case-count (1,939). Also, *Salmonella* spp. caused almost double the number of single etiology outbreaks as recorded in the previous 10 years (30%) (Dewey-Mattia et al., 2018). Annually, non-typhoidal *S. enterica* illnesses are estimated to cost approximately 3.3 billion U.S. dollars, more than any other of 14 major foodborne pathogens (Hoffmann et al., 2012).

## Routes of Fresh Produce Contamination

Fresh produce is generally minimally processed (e.g., washed, sanitized, packaged) and therefore does not go through additional processing steps that would further reduce the microbiological burden and eliminate harmful pathogens (e.g., heating). Since fresh produce is often grown underground or near the ground, it is expected that the saprophytic environmental microorganisms that inhabit soil, water, and vegetation would also be present on these food items. Previous mesophilic evaluation of bacterial levels on fresh produce showed 10^3^–10^9^ CFU/g before and 10^3^–10^6^ CFU/g after minimal processing (Nguyen-the and Carlin, 1994). It is estimated that 80–90% of the mesophilic bacteria are Gram-negative rods including *Enterobacter* spp., *Erwinia* spp., and *Pseudomonas* spp. (Magnuson et al., 1990; Marchetti et al., 1992; Nguyen-the and Carlin, 1994). Yeast and mold levels on fresh produce range from 10^2^ to 10^6^ (Garg et al., 1990; King et al., 1991; Nguyen-the and Carlin, 1994). Even with the high population levels of the native microflora, the quality of fresh produce is often still acceptable.

Due to minimal processing, the concern associated with fresh produce consumption is the possibility for contamination with foodborne pathogens. For example, cantaloupe is grown close to the ground, and the melons can come into contact with soil, other vegetation, irrigation water, and fertilizer. Studies assessing the presence of *S. enterica* on cantaloupes have observed the prevalence of the pathogen in samples as low as 0.76% (11/1440) (Madden, 1992) and as high as 5.3% (8/151) (FDA, 1999). In addition to pre-processing contamination, fresh produce can also become contaminated with foodborne pathogens during harvesting and various post-harvest stages including processing, transport, and storage.

### Pre-harvest Contamination

Prior to harvest, fresh produce can become contaminated with foodborne pathogens from the environment. Pathogens can be introduced into the environment via irrigation water, soil, fertilizers, manure, insects, and dust. Irrigation water is a known vector for foodborne pathogen contamination, and its quality is an indicator of produce safety. Natural sources of irrigation water include rivers, collected rainwater, aquifers, and groundwater (Uyttendaele et al., 2015). Irrigation water can also come from the reuse of wastewater, which could potentially be contaminated with human pathogens depending on how the water was treated. One study found fecal coliform counts as high as 4 log CFU/ml in irrigation water (Materon et al., 2007). Irrigation water can effectively transmit *E. coli* O157:H7 to row crops such as lettuce (Solomon et al., 2002; Wachtel et al., 2002a); and epidemiological investigations have traced foodborne pathogen contamination of fresh produce to irrigation water (Ackers et al., 1998; Herwaldt, 2000; Wachtel et al., 2002b). For example, irrigation water sourced from a pond was responsible for contaminating tomatoes that led to a multistate *S. enterica* outbreak in 2005 (Greene et al., 2005).

Soil, fertilizers, and animal manure can also be potential sources for fresh produce contamination. Saprophytic bacteria, such as *L. monocytogenes*, are ubiquitous in soil and can readily contaminate produce grown underground or low to the ground. Soil amendments, such as fertilizer, compost, and manure can introduce or spread foodborne pathogens to crops. Farm animals such as cows, sheep, pigs, chickens, and deer shed pathogenic *E. coli* and *S. enterica* species in their feces, with prevalence in fecal samples as high as 70% (Bailey et al., 2001; Dargatz et al., 2003; Hussein and Bollinger, 2005; Keen et al., 2006; Doane et al., 2007). Fresh produce can be exposed to these pathogens and other enteric pathogens through nearby farm or wildlife animals, along with water runoff containing animal feces. In 2006, feral swine feces was thought to be responsible for the contamination of agricultural and surface water at a ranch which led to an outbreak of *E. coli* O157:H7 associated with bagged spinach in the U.S. (26 states involved) and Canada; 205 illnesses and three deaths were reported (Jay et al., 2007).

Air, dust, insects, and invertebrates are also vectors for the transfer of foodborne pathogens to fresh produce. Airborne transmission of *E. coli* O157:H7 from a cattle feedlot to leafy greens fields 60–180 m away has been demonstrated experimentally (Berry et al., 2015). Wildlife that roams and forages in fields may shed foodborne pathogens in fecal matter. Local and migratory birds may also serve as vectors for foodborne pathogens as they frequent fields to obtain food and shelter (Cernicchiaro et al., 2012). In addition, nematodes, slugs, and insects have been shown to harbor bacterial pathogens such as *E. coli* O157:H7, *Campylobacter*, *Shigella*, *S. enterica*, and *L. monocytogenes* (Kenney et al., 2006; Sproston et al., 2006; Khamesipour et al., 2018). Insects can facilitate the contamination of fresh produce, for example, various species of flies are capable of transferring *E. coli* O157:H7 to spinach leaves (Talley et al., 2009; Wasala et al., 2013) and apples (Janisiewicz et al., 1999) through their excreta or regurgitation.

### Contamination During Harvesting

Contamination of fresh produce with foodborne pathogens can occur during harvesting through contact with contaminated equipment, transport containers, knives and tools, and human hands and gloves. Mechanical harvesting equipment, including vegetable cutters and corers, can harbor pathogens and disseminate these organisms to fresh produce during harvesting (McEvoy et al., 2009; Taormina et al., 2009; Yang et al., 2012). Leafy greens are often cut and cored in the field by a field-core harvesting technique. During simulated field harvesting, iceberg lettuce was cross-contaminated with *E. coli* O157:H7 when the pathogen was on the blade of the field coring device (Taormina et al., 2009). A contaminated field coring knife is capable of transferring *E. coli* O157:H7 to as many as 19 heads of lettuce (McEvoy et al., 2009).

It is estimated that 20% of all foodborne bacterial illnesses are due to food worker transmissions (Greig et al., 2007). In the U.S. during the 2000s, a total of 647 foodborne outbreaks, causing 54,888 illnesses, were attributed to food worker transmissions (Greig et al., 2007). Out of these outbreaks, 23% (151) were due to the consumption of food contaminated with *S. enterica*; of which 5% (7) were associated with produce and lead to a total of 1263 illnesses. Food worker pathogen transmission to food products may be due to poor hygienic conditions (personnel or environmental), working while sick, or due to improper food safety training and good agricultural practices. Food workers may also be asymptomatic carriers of foodborne pathogens, such as *S. enterica* Typhi. Various studies have determined that pathogens are transmissible from humans to food surfaces (Jimenez et al., 2007; Sharps et al., 2012; Brar and Danyluk, 2013). For example, *S. enterica* has been documented to be transmitted from gloves to tomatoes during harvesting in a laboratory setting (Brar and Danyluk, 2013). Proper training for food handlers, including frequent glove changes and handwashing, is essential to prevent the spread of foodborne pathogens.

### Post-harvest and Processing Contamination

Post-harvest contamination of fresh produce can occur during transport, storage, and processing. Once harvested from the field, produce is chilled and transported while refrigerated to its next destination. Since rapid cooling of produce is essential to minimize product damage and increase shelf life, coolers, ice or water cooling, and transport vehicles can be possible sources of pathogen contamination. Fresh produce is often transported in a highway trailer either on ice (as is used with broccoli, green onions, and sweet corn) or with a mechanical cooling system (used with lettuce and other leafy greens) (Suslow et al., 2003). In addition to maintaining optimal quality and respiration, strict temperature control of fresh produce is needed to reduce the risk associated with illness due to foodborne pathogens. After transport and once at a processing facility, fresh produce can be stored in a packing house for as little as a few hours or up to several days.

Fresh produce is generally minimally processed with stages that include washing/fluming, shredding and cutting, drying, and packaging. Produce contamination can occur due to contaminated equipment, or due to cross-contamination with other produce. Many studies have assessed the contamination or cross-contamination of produce items during processing (Buchholz et al., 2012a, b, 2014; Davidson et al., 2017; Smolinski et al., 2018). For example, both *S. enterica* and *E. coli* O157:H7 have been shown to cross-contaminate spinach, cilantro, and Romaine lettuce during pilot plant-scale processing including washing/fluming (Smolinski et al., 2018). Cross-contamination of pathogens can also occur from processing equipment to food products. A 33.3% (30) frequency of *E. coli* was observed from environmental samples of a conveyor system at a melon farm in Texas (Castillo et al., 2004). Once washed and packaged, produce can become contaminated during storage if the food is stored in a contaminated location, contaminated box, or enclosure. A 0.8 and a 1.6% (250) frequency of isolation of *S. enterica* and *E. coli*, respectively, on washed cantaloupe melons stored in a cooler within a cardboard box at the same melon farm has also been observed (Castillo et al., 2004). The melons could have become contaminated from the field, during harvest or processing, or during storage in the cardboard box.

Once minimally processed, fresh produce is distributed to retail food establishments, food service facilities, and restaurants. Handling of fresh produce by food service personnel, along with the cutting and chopping of fresh produce are all scenarios where these foods can become contaminated. In addition, display temperatures above refrigeration at retail can increase the risk of illness due to possible pathogen proliferation. Before use for cutting or chopping of fresh produce items, equipment including knives and cutting boards should be sanitized. Fresh produce should also be segregated from raw meat and poultry. A knife can cross-contaminate lettuce with *S. enterica* if it is first used to cut raw chicken (Ravishankar et al., 2010). Once cut, fresh produce is often packaged in deli containers or plastic wrap and is stored in retail display cabinets. Proper refrigeration temperature should be maintained to reduce the risk of bacterial pathogen proliferation. For example, the growth rates of *L. monocytogenes* were significantly higher on fresh-cut produce items stored in deli containers at 10°C when compared to 5°C (Salazar et al., 2017).

## Multistate Outbreaks of Foodborne Illness Associated With Fresh Produce

Data were obtained from the National Outbreak Reporting System (NORS), which tracks foodborne disease outbreaks from public health departments in the U.S (CDC, 2017e). This database does not include outbreaks caused by an exposure event that occurred outside of the U.S. and is limited to outbreaks that are detected, investigated, and reported. Outbreaks considered for inclusion in this review must have resulted in foodborne enteric illness due to a confirmed pathogen in more than one state in the U.S between 2010 and 2017. The implicated food vehicle in each outbreak also must have been classified as produce based on the Food Safety Analytics Collaboration categories (IFSAC, Richardson et al., 2017). In this categorization method, one food category is assigned when the implicated food has a single ingredient and when each ingredient in the food belongs to the same food category. In this review, outbreaks were excluded if they were linked to a food that had multiple ingredients unless the ingredients were all classified within the same category. Outbreaks were also excluded if the implicated food vehicle was dried or frozen. A case study is presented in section A Case Study: Caramel Apples, which is not included in the outbreak tally of this review as it does not meet the inclusion criteria but is discussed as the unique characteristics of the outbreak highlight issues related to multi-component food products that contain fresh produce.

Overall, 85 multistate outbreaks associated with fresh produce with confirmed etiologies occurred in the U.S. from 2010 to 2017. A total of five pathogens were responsible for these outbreaks; 83 outbreaks were attributed to three bacterial pathogens, while the remaining two outbreaks were linked to Hepatitis A and *Cyclospora cayetanensis*. As bacterial pathogens were the principal cause of multistate outbreaks associated with fresh produce in the time period of interest, this review will focus on these organisms. *S. enterica* was linked to over half of the multistate outbreaks due to bacterial pathogens (67.5%) with 32 different confirmed serotypes. The most frequent single-etiology outbreaks of *S. enterica* infection were attributable to serotypes Newport (10), Enteritis (6), and Javiana (5). Pathogenic *E. coli* was responsible for 27.4% of the outbreaks, and *L. monocytogenes* was responsible for 4.8%. These outbreaks resulted in a known combined total of 4658 illnesses, 1187 hospitalizations, and 55 deaths. *S. enterica* was the cause of the majority of known illnesses (81.1%) and known hospitalizations (66.2%), however, *L. monocytogenes* was responsible for the majority of known deaths (67.3%), which were mostly from a single outbreak (33 known deaths; see section Cantaloupe). Vegetables were implicated in 69.9% of outbreaks, and the remainder were linked to fruits (Table 1); however, more deaths occurred due to outbreaks associated with fruits (76.4%) as compared to vegetables (24.6%). The most frequently identified food vehicles within the vegetable category were sprouts (27.6%) and lettuce (13.8%), followed by cucumbers, Romaine, and leafy greens (12.1% each), but outbreaks associated with cucumbers resulted in nearly half (42.8%) of all illnesses linked to vegetables. Melons were the dominant food vehicle for bacterial outbreaks in the fruit category (40.0%) regarding known illnesses (44.9%), hospitalizations (60.0%), and deaths (90.5%).

**TABLE 1:** Multistate foodborne outbreaks of bacterial infection in the U.S. from 2010 to 2017 associated with different fresh produce categories.

**Fresh produce**	**Multistate**	**Illnesses**	**Hospitalizations**	**Deaths**
**category**	**outbreaks *n* = 83**	***n* = 4501**	***n* = 1117**	***n* = 55**
Fruits (total)	25	1443	530	42
Melons	10	578	276	38
Papaya	7	418	113	3
Mango	3	181	49	0
Avocado	1	59	7	0
Grapes	1	27	10	0
Coconut	1	14	2	0
Stone fruits	1	2	2	1
Unspecified fruit	1	7	1	0
Vegetables (total)	58	3215	657	13
Sprouts	16	603	99	3
Lettuce	8	144	43	1
Cucumber	7	1375	297	7
Leafy greens^a^	7	181	58	2
Romaine	7	358	100	0
Tomatoes	6	434	35	0
Spinach	3	22	4	0
Peppers	2	53	13	0
Onions	1	29	6	0
Cabbage	1	16	2	0

**^*a*^Includes leafy greens not categorized as lettuce, Romaine, or spinach.**

### Pathogenic *E. coli*

A total of 23 multistate outbreaks were attributed to pathogenic *E. coli* and associated with fresh produce between 2010 and 2017 (Table 2). These outbreaks caused a combined total of 550 illnesses, and *E. coli* O157:H7 was responsible for over half of these cases (377). Vegetable row crops such as lettuce were identified as food vehicles in the majority of these outbreaks (87.0%), and the remainder were linked to sprouts. No outbreaks were linked to fruits. Six total *E. coli* serotypes were implicated in these outbreaks, and no single outbreak had more than one confirmed etiology. *E. coli* O157:H7 was the dominant serotype, responsible for 13 out of the 23 outbreaks (56.5%), of which 100% were associated with vegetable row crops.

**TABLE 2:** Multistate foodborne outbreaks in the U.S. from 2010 to 2017 due to *Escherichia coli* associated with fresh produce.

**Year**	**Fresh produce**	**Associated *E. coli***	**Total # of**	**Illnesses**	**Hospitalizations**	**Deaths**	**References (in addition**
	**category**	**serotype**	**states**		**[total (% of illnesses^a^)]**	**[total (% of illnesses^b^)]**	**to NORS database)**
2010	Romaine	O145	5	31	14	0 (0)	CDC, 2010a
2011	Romaine	O157:H7	9	60	35 (67.3)	0 (0)	Slayton et al., 2011; CDC, 2012a, e
	Sprouts	O26	11	29	7 (38.9)	0 (0)	FDA, 2012b
	Lettuce	O157:H7	NA	26	5 (29.4)	0 (0)	
2012	Romaine	O157:H7	NA	52	NA	NA	
	Leafy greens	O157:H7	5	33	13 (46.4)	0 (0)	CDC, 2012d
	Lettuce	O157:H7	3	24	NA	NA	Marder et al., 2014
	Lettuce	O145	NA	16	6 (37.5)	0 (0)	
	Spinach	O157:NM (H-)	NA	10	NA	NA	
2013	Romaine	O157:H7	4	33	9 (28.1)	0 (0)	FDA, 2013b
	Lettuce	O26	NA	26	5 (23.8)	0 (0)	
	Leafy greens	O157:H7	NA	14	9 (81.8)	1 (9.1)	
	Sprouts	O121	6	19	5 (26.3)	0 (0)	CDC, 2014
2014	Cabbage	O111	NA	16	2 (100)	0 (0)	
	Lettuce	O157:H7	NA	11	2 (22.2)	0 (0)	
	Spinach	O157:H7	NA	4	1 (25.0)	0 (0)	
2015	Leafy greens	O145	NA	7	5 (71.4)	0 (0)	
2016	Sprouts	O157:NM (H-)	2	11	2 (18.2)	0 (0)	CDC, 2016d
	Lettuce	O157:H7	NA	11	4 (36.4)	0 (0)	
2017	Leafy greens	O157:H7	NA	68	18 (26.5)	0 (0)	
	Leafy greens	O157:H7	15	25	9 (36.0)	1 (4.0)	CDC, 2018
	Spinach	O26	NA	8	3 (37.5)	0 (0)	

**^*a*^% of illnesses was calculated based on available hospitalization information in the NORS database. ^*b*^% of deaths was calculated based on available death information in the NORS database. NA, defined as multistate in NORS database, information on the number of states involved was not available in the NORS database or CDC website.**

#### Romaine Lettuce

Between 2010 and 2017, five outbreaks of *E. coli* infection occurred that were associated with Romaine lettuce. Four out of these five outbreaks were linked to *E. coli* O157:H7 and caused a total of 192 illnesses and no known deaths. An outbreak in 2011 affected the largest number of states (9) and resulted in the largest number of illnesses (58) (Slayton et al., 2011; CDC, 2012a) of all *E. coli* outbreaks due to contaminated Romaine lettuce in this timeframe. Illness onset occurred from October 9, 2011, to November 7, 2011, and 33 hospitalizations, three cases of HUS, and no deaths were reported (CDC, 2012a). The majority of cases occurred in Missouri (65.5%) and were female (59%). The median age of cases was 28 years, yet the age range was 1–94 years. Epidemiological data pointed to Romaine lettuce as the food vehicle in this outbreak, and one farm was identified as the likely source of the contaminated product, but confirmation via environmental testing was not obtained (Slayton et al., 2011).

#### Other Leafy Greens

A total of 15 outbreaks of *E. coli* infection occurred between 2010 and 2017 that were associated with vegetable row crops other than Romaine lettuce (i.e., spinach, iceberg lettuce). These outbreaks resulted in a total of 299 illnesses and two known deaths. Nine of these outbreaks were associated with *E. coli* O157:H7 infection (60.0%), and this pathogen was the cause of both of the known deaths. In 2011, an outbreak of *E. coli* O157:H7 infection associated with an organic spinach and spring mix salad blend occurred that caused 33 cases in five states (CDC, 2012d). This outbreak was associated with the most illnesses due to the consumption of contaminated vegetable row crops other than Romaine lettuce that took place in a private residential setting. A total of 13 hospitalizations and no known deaths occurred, but two cases of HUS were reported. Illness onset took place from October 18, 2012 to November 12, 2012, and New York state reported 78.8% of the cases. The ages of cases ranged from four to 66, with a median age of 24, and 63% were female. Epidemiological data and laboratory testing of left-over organic spinach and spring mix blend identified pre-packaged leafy greens produced in Massachusetts as the food vehicle of the outbreak, however, the source of the *E. coli* O157:H7 contamination was not determined.

#### Sprouts

Three multistate outbreaks of *E. coli* associated with sprouts occurred between 2010 and 2017. All outbreaks originated from foods consumed in the restaurant setting and affected a range of six to 11 states (based on available CDC data). Clover sprouts were implicated in two of the three outbreaks, while alfalfa sprouts were implicated in the remaining outbreak. The largest of these outbreaks occurred between 2011 and 2012 and was due to *E. coli* O26 contamination of clover sprouts (CDC, 2012e; FDA, 2012b). A total of 29 illnesses were reported that resulted in seven hospitalizations and zero deaths with illness onset from December 25, 2011 to March 3, 2012. Out of the 11 affected states, the highest case counts were reported from Michigan (10) and Iowa (5). The majority of cases were female (89%), and the median age was 26 years, with a range of nine to 57 years of age (CDC, 2012e). Traceback analysis revealed that clover sprouts were the food vehicle in this outbreak, which were likely produced from contaminated seeds (FDA, 2012b).

### L. monocytogenes

Between 2010 and 2017, four multistate listeriosis outbreaks associated with fresh produce occurred in the U.S (Table 3). A total of 173 illnesses occurred due to these outbreaks, which led to a 99.4% known hospitalization rate and a 21.8% known death rate. Two of these four multistate outbreaks implicated fruits, while vegetable row crops and sprouts were confirmed food vehicles in the remaining two outbreaks.

**TABLE 3:** Multistate foodborne outbreaks in the U.S. from 2010 to 2017 due to *Listeria monocytogenes* associated with fresh produce.

**Year**	**Fresh produce category**	**Associated *L. monocytogenes* serotype(s)**	**Total # of states**	**Illnesses**	**Hospitalizations [total (% of illnesses^a^)]**	**Deaths [total (% of illnesses^b^)]**	**References**
2011	Cantaloupe	1/2a, 1/2b	28	147	143 (99.3)	33 (22.9)	CDC, 2011d; Laksanalamai et al., 2012; McCollum et al., 2013
2014	Sprouts		2	5	5 (100.0)	2 (40.0)	CDC, 2015c
	Stone fruits	4b	2	2	2 (100.0)	1 (50.0)	Jackson et al., 2015; Chen et al., 2017
2016	Lettuce		9	19	19 (100.0)	1 (5.3)	Self et al., 2016

**^*a*^% of illnesses was calculated based on available hospitalization information in the NORS database. ^*b*^% of deaths was calculated based on available death information in the NORS database. NA, defined as multistate in NORS database, information on the number of states involved was not available in the NORS database or CDC website.**

#### Cantaloupe

A total of 147 listeriosis cases were reported to the CDC between July and October 2011, which resulted in 33 known deaths (CDC, 2011d; McCollum et al., 2013). Of the cases with available data, 143 persons out of 145 were hospitalized, three illnesses were in newborns, and four illnesses were in pregnant women, one of which resulted in a miscarriage. Overall, cases were reported in 28 states, with the most cases from Colorado (40), Texas (18), and New Mexico (15). Of patients questioned, 134 out of 144 reported the consumption of cantaloupe within the month before illness. Low sanitation and improper processing equipment were later determined to have been the likely cause of cantaloupe contamination at the implicated farm.

Five subtypes of *L. monocytogenes* were involved in this outbreak, all of which were isolated from whole cantaloupes and environmental samples that originated from one Colorado-based farm. Initially, three subtypes and the attributable cases were identified through PulseNet (CDC, 2017g). Later, two more subtypes were identified via food and environmental samples, which linked two additional cases. Both *L. monocytogenes* serotypes 1/2a and 1/2b were implicated in this outbreak (Laksanalamai et al., 2012). Since this outbreak, follow-up studies have been conducted to assess the growth and survival of *L. monocytogenes* on whole and fresh-cut cantaloupe melons (Ukuku et al., 2012; Huang et al., 2015; Nyarko et al., 2016a, b; Salazar et al., 2017). For example, the growth rate of *L. monocytogenes* on cantaloupe rind and cut cantaloupe flesh has been modeled at various storage temperatures (Danyluk et al., 2014; Salazar et al., 2017).

#### Lettuce

Lettuce contaminated with *L. monocytogenes* caused 19 illnesses in nine states between July 2015 and January 2016 (Self et al., 2016). All cases were hospitalized, one illness was pregnancy related, and one death was reported. Of people who were interviewed, 13 out of 14 reported consuming packaged salad, and nine indicated a specific brand. Routine sampling by the Ohio Department of Agriculture identified *L. monocytogenes* in the specified brand of packaged salad obtained from a retail location. Whole genome sequencing (WGS) confirmed a high degree of genetic similarity between patient and food isolates. A voluntary recall of packaged salads made at the facility that produced the implicated packaged salad occurred in late January 2016.

#### Stone Fruits

In July 2014, a packing company recalled various types of stone fruits including whole peaches and nectarines due to suspected *Listeria* contamination (Jackson et al., 2015). Four patient isolates in PulseNet (CDC, 2017g) matched two pulse-field gel electrophoresis (PFGE) patterns produced by *L. monocytogenes* isolated from implicated stone fruit once the data were uploaded in August. WGS later confirmed that two of the four isolates from patients in Massachusetts and Minnesota were highly related to outbreak strains. Both confirmed patient isolates were *L. monocytogenes* serotype 4b (Chen et al., 2017).

#### Sprouts

The FDA identified *L. monocytogenes* contamination in mung bean sprouts and irrigation water during routine inspection of a production facility in August 2014. A subsequent investigation cited poor sanitation and equipment maintenance. The company issued a voluntary recall; however, a follow-up inspection demonstrated continued environmental contamination, and the company has since closed its production facility. PFGE and WGS indicated five possible cases of listeriosis associated with this outbreak in Illinois (4) and Michigan (1). All cases were hospitalized, and two deaths were reported (CDC, 2015c). Both interviewed patients indicated consumption of bean sprouts within the month before they became ill.

#### A Case Study: Caramel Apples

Between October 2014 and January 2015, 35 illnesses from 12 states were reported to the CDC due to consumption of *L. monocytogenes*-contaminated prepackaged caramel apples (CDC, 2015a). This outbreak warrants discussion as the multi-commodity product consumed (i.e., caramel apple) had a fresh produce component (i.e., whole apple) that was considered low risk because apples have a low internal pH that does not support the growth of pathogens. In addition, the low water activity and temperature of the hot caramel coating applied to the whole apple during processing would hinder pathogen survival if present on the outside surface of the apple. In all, 34 individuals were hospitalized, and 11 cases of listeriosis occurred in pregnant women or newborn infants, including one case that resulted in fetal loss. A total of seven deaths were associated with this outbreak, and at least three were attributable to listeriosis. Three instances of meningitis were reported in healthy children from five to 15 years of age. One individual was determined to be co-infected with two strains of *L*. *monocytogenes.*

Although two separate outbreak clusters were identified via both PFGE and WGS, they were investigated together due to the co-infected patient and the comparable regionality and temporality of the clusters. Interviews of 31 infected individuals revealed that 28 had consumed commercially produced prepackaged caramel apples in the month before illness. The remaining three infected individuals interviewed reported consumption of whole or sliced green apples, however, it is unknown if these apples were a food vehicle in the outbreak. *Listeria* contamination present in an apple packing facility that supplied apples to the implicated caramel apple manufacturers was determined by the FDA to be highly related to the outbreak strains via both PFGE and WGS methods. Follow-up research indicated that stick insertion into the core of the apple creates a microenvironment that allows *L. monocytogenes* to survive and even grow in an otherwise unsupportive food vehicle (Glass et al., 2015; Salazar et al., 2016). A novel approach for the control of *L. monocytogenes* in caramel apples through the pretreatment of the wooden sticks with potassium sorbate has since been proposed (Carstens et al., 2018).

### S. enterica

*S. enterica* was the reported etiologic agent for a total of 56 multistate outbreaks associated with fresh produce between 2010 and 2017 (Table 4). A total of 3778 illnesses were linked to these outbreaks, which resulted in a 28.3% known hospitalization rate and 16 known deaths. Fruits were the majority food vehicle implicated in outbreaks attributed to *S. enterica* (41.1%), followed by seeded vegetables (26.8%), sprouts (21.4%), vegetable row crops (8.9%), and root-underground vegetables (1.8%). However, seeded vegetables (49.3%) rather than fruits were the dominant food vehicle implicated in illnesses due to *S. enterica* infection (30.1%).

**TABLE 4:** Multistate foodborne outbreaks in the U.S. from 2010 to 2017 due to *Salmonella enterica* associated with fresh produce.

**Year**	**Fresh produce category**	**Associated *S. enterica* serotype(s)**	**Total # of states**	**Illnesses**	**Hospitalizations [total (% of illnesses^a^)]**	**Deaths [total (% of illnesses^b^)]**	**References (in addition to NORS database)**
2010	Sprouts	I 4,[5],12:i:-	26	140	31(*NA*)	0(*NA*)	CDC, 2011c
	Tomato	Newport	NA	64	NA	NA	
	Sprouts	Newport	11	44	NA	NA	CDC, 2010b
	Tomato	Javiana	NA	30	8(*NA*)	NA	
	Tomato	Newport	NA	24	NA	NA	
	Sprouts	Newport	NA	9	NA	NA	
	Sprouts	Cubana	NA	3	NA	NA	
2011	Tomato	Newport	NA	166	0 (0)	0 (0)	
	Papaya	Agona	25	106	10 (9.6)	0 (0)	CDC, 2011a
	Sprouts	Enteritidis	5	27	3 (11.1)	0 (0)	CDC, 2011b
	Cantaloupe	Uganda	NA	25	4 (16.0)	0 (0)	
	Cantaloupe	Panama	10	20	3 (21.4)	0 (0)	CDC, 2011e
2012	Cantaloupe	Typhimurium, Newport	24	261	94 (36.1)	3 (1.1)	CDC, 2012c
	Mango	Braenderup, Worthington	15	129	33 (32.7)	0 (0)	CDC, 2012b
	Cucumber	Javiana	NA	49	14 (35.0)	0 (0)	
	Cantaloupe	Newport	NA	33	11 (42.3)	1 (3.8)	
	Cantaloupe	Newport	NA	24	5 (33.0)	0 (0)	
	Sprouts	Cubana	3	19	0 (0)	0 (0)	FDA, 2012c
	Romaine	Newport	NA	15	1 (100.0)	0 (0)	
	Cantaloupe	Typhimurium	NA	14	1 (16.7)	0 (0)	
2013	Tomato	Saintpaul	NA	131	23 (26.4)	0 (0)	
	Cucumber	Saintpaul	18	84	17 (28.3)	0 (0)	CDC, 2013
	Papaya	Thompson	NA	13	6 (46.2)	1 (7.7)	
	Unspecified fruit	Virchow	NA	7	1 (14.3)	0 (0)	
2014	Cucumber	Newport	29	275	48 (34.0)	1 (0.4)	Angelo et al., 2014
	Sprouts	Enteritidis	12	115	19 (25.3)	0 (0)	CDC, 2015b
	Cucumber	Javiana	NA	36	8 (25.0)	0 (0)	
	Grapes	Saintpaul	NA	27	10 (28.5)	0 (0)	
	Peppers	Paratyphi B	NA	21	5 (26.3)	0 (0)	
	Cantaloupe	Baildon	NA	20	6 (35.3)	0 (0)	
	Mango	Minnesota	NA	4	1 (25.0)	0 (0)	
2015	Cucumber	Poona	40	907	204 (28.3)	6 (0.8)	CDC, 2016b
	Sprouts	Muenchen, Cubana, Kentucky	12	32	10 (32.3)	1 (3.2)	CDC, 2016a
	Tomato	Hartford	NA	19	4 (33.3)	0 (0)	
	Lettuce	Paratyphi B	NA	11	2 (20.0)	0 (0)	
2016	Avocado	Enteritidis	19	59	7 (17.1)	0 (0)	CDC, 2017f
	Sprouts	Ready, Abony	9	36	7 (20.6)	0 (0)	CDC, 2016c
	Peppers	Anatum	9	32	8 (32.0)	0 (0)	Hassan et al., 2017
	Sprouts	Braenderup	NA	32	6 (24.0)	0 (0)	
	Onions	Javiana	NA	29	6 (20.7)	0 (0)	
	Sprouts	Enteritidis	NA	20	3 (21.4)	0 (0)	
	Cucumber	Oslo	8	14	3 (23.1)	0 (0)	Bottichio et al., 2016
	Cantaloupe	Minnesota	NA	10	3 (24.9)	0 (0)	
	Cucumber	Saintpaul	NA	10	3 (30.0)	0 (0)	
	Leafy greens	Enteritidis	NA	7	0 (0)	0 (0)	
2017	Papaya	Agona, Gaminara, Kiambu, Thompson, Seftenberg	23	213	68 (31.9)	1 (0.5)	CDC, 2017a
	Romaine	Enteritidis	36	151	31 (20.5)	0 (0)	Clark, 2017
	Sprouts	Montevideo, Cubana	NA	62	3 (4.8)	0 (0)	
	Papaya	Braenderup	NA	55	18 (32.7)	0 (0)	
	Mango	Infantis	NA	48	15 (31.3)	0 (0)	
	Leafy greens	Javiana	NA	27	4 (14.8)	0 (0)	
	Melon	Newport	2	24	6 (25.0)	1 (4.2)	FSN, 2017
	Coconut	Chailey	7	14	2 (14.3)	0 (0)	Luna et al., 2017
	Papaya	Urbana	3	7	4 (57.1)	0 (0)	CDC, 2017c
	Papaya	Infantis, Newport	4	4	2 (50.0)	0 (0)	CDC, 2017b

**^*a*^% of illnesses was calculated based on available hospitalization information in the NORS database. ^*b*^% of deaths was calculated based on available death information in the NORS database. NA, information not available in the NORS database or CDC website.**

#### Cucumbers

Of all multistate outbreaks of *S. enterica* associated with seeded vegetables from 2010 to 2017, 46.7% were linked to cucumbers. These outbreaks resulted in a combined 1375 illnesses, 297 known hospitalizations, and seven known deaths. *S. enterica* serotypes Saintpaul (2) and Javiana (2) were most frequently confirmed etiologies in these outbreaks; however, Newport (1), Poona (1), and Oslo (1) were also implicated. The largest outbreak in terms of both the number of states affected (40) and case count (907) occurred in 2015 and was linked to cucumbers imported from Mexico that were contaminated with *S. enterica* Poona (CDC, 2016b). The majority of cases were female (56%) and under 18 years of age (49%) with illness onset from July 3, 2015, to February 29, 2016. A total of 204 hospitalizations and six deaths were reported as a result of this outbreak. A common distributor of cucumbers from Mexico was linked to this outbreak through testing of cucumbers at retail locations as well as at the distributor’s facility. Two voluntary recalls in September 2015 resulted, after which the number of new cases decreased. Although recalled cucumbers should no longer have been available in stores, 127 cases with illness onset after September 24 were reported, which are suspected to have resulted from cross-contamination during shipping or at the retail level.

#### Melons

Nine outbreaks of *S. enterica* infection associated with melons occurred between 2010 and 2017, which comprised 39.1% of all *S. enterica* outbreaks linked to fruits. Of these melon-attributable outbreaks, eight implicated cantaloupe and the other an unspecified type of melon. The most frequent *S. enterica* serotype involved in single-etiology outbreaks linked to melons was Newport (3), which was also involved in the only multi-etiology outbreak associated with melons along with Typhimurium. These melon-associated outbreaks collectively caused a total of 431 illnesses, 133 known hospitalization, and five deaths.

Cantaloupe was the food vehicle implicated in the largest outbreak of salmonellosis by case count linked to any fruit type during this period (CDC, 2012c). In 2012, 261 illnesses occurred across 24 states that resulted in a 36.0% hospitalization rate and three deaths. The highest number of illnesses occurred in Kentucky (66) followed by Illinois (36) and Indiana (30); however, all three deaths occurred in Kentucky. Illness onset occurred between July 6 and September 16, 2012, and cases were primarily female (55%) with a median case age of 47 years. Epidemiological data and traceback analysis identified one farm as the source of the contaminated cantaloupes (FDA, 2013a). Environmental samples from the farm’s facilities and cantaloupes obtained at the farm matched the PFGE patterns of the *S. enterica* Typhimurium and Newport strains identified in clinical isolates; the *S. enterica* Typhimurium outbreak strain was also isolated from cantaloupes at the retail level in Kentucky. A recall of all implicated cantaloupes was announced in August 2012, and it was ultimately determined that poor agricultural and sanitation practices at the implicated farm contributed to the cause of this outbreak (FDA, 2012a).

#### Other Fruits

A total of 14 outbreaks due to *S. enterica* linked to fruits other than melons occurred between 2010 and 2017. Papaya was the most frequent food vehicle implicated in these outbreaks (7), followed by mango (3). Between three and 25 states were affected by these outbreaks (CDC, 2011a,e, 2012b, 2017a,b,c; FDA, 2013a), and an assortment of 17 *S. enterica* serotypes were implicated, with up to five serotypes confirmed in a single outbreak. These fruit-associated outbreaks resulted in a combined total of 706 illnesses, 182 known hospitalizations, and three known deaths.

The largest outbreak of salmonellosis due to case count in this category was associated with imported papaya and involved 23 states (CDC, 2017a). This outbreak was also the largest of four multistate outbreaks that occurred in 2017 associated with papaya contaminated with *Salmonella* spp. A total of 213 illnesses, 68 known hospitalizations, and one death occurred as a result of this outbreak, with illness onset between May 17, 2017, and October 4, 2017. The median case age was 40, but ages ranged from one to 95. New York reported the majority of cases (71) along with the only death linked to this outbreak, and New Jersey (41) and Virginia (18) reported the second and third most cases. Traceback analysis pointed toward papaya as the food vehicle in this outbreak, and product sampling of papaya from a grocery store by the Maryland Department of Health yielded two of the five *S. enterica* serotypes implicated. WGS and PFGE methods were used to confirm the implicated serotypes, and Thompson was linked to the majority of cases (144). A single farm in Mexico was the ultimate source of the contaminated papayas, and three recalls of papayas resulted. Recall dates spanned from July 26 to August 7, 2017, however, the outbreak did not end for another 2 months.

#### Hot Peppers

Two outbreaks between 2010 and 2017 were linked to *S. enterica*-contaminated peppers, which constituted 13.3% of all outbreaks associated with seeded vegetables during this period. In 2014, *S. enterica* serotype Paratyphi B was the confirmed etiology of an outbreak linked to mini peppers, while in 2016, a slightly larger outbreak resulted due to hot peppers contaminated with *S. enterica* Anatum. The later outbreak caused 32 illnesses, eight known hospitalizations, and zero deaths across nine states (Hassan et al., 2017). Illness onset occurred between May 6 and July 9, 2016, and cases ranged from four to 79 years of age, of which 59% were female. The consolidation of peppers from various sources before distribution and the inability of all patients to identify the type of pepper they had consumed before illness prevented the confirmation of a specific variety of hot pepper as the food vehicle in this outbreak. WGS revealed that 19 clinical isolates were highly correlated to an isolate from a contaminated Anaheim pepper, however, this could not be confirmed by epidemiological data.

#### Sprouts

Between 2010 and 2017, 12 multistate outbreaks linked to sprouts occurred as a result of *S. enterica* contamination that affected a range of three to 26 states (CDC, 2011b,c, 2015b, 2016a,c; FDA, 2012c). These outbreaks primarily occurred in a restaurant setting and caused a collective 539 illnesses, 82 known hospitalizations, and one known death. Ten different serotypes of *S. enterica* were implicated in these outbreaks, with up to three serotypes associated with a single outbreak (CDC, 2011c). The largest outbreak associated with sprouts contaminated with *S. enterica* occurred in 2010 and caused 140 illnesses in 26 states. Illness onset took place from November 1, 2010, to February 9, 2011, and 31 known hospitalizations were reported along with zero deaths. The majority of illnesses occurred in Illinois (70), Missouri (23), and Indiana (13) and the remaining states involved reported four or fewer cases each. The median case age was 28 years, with a range from one to 85 years, and 63% were female. Several cases consumed contaminated alfalfa sprouts contained in sandwiches from a single fast food restaurant chain. Environmental sampling of the implicated farm identified the confirmed outbreak strain of *S. enterica* I 4,[5],12:i:- in water run-off, but not in product samples.

The second largest outbreak linked to sprouts contaminated with S. *enterica* occurred in 2014 and affected 12 states with 115 cases (CDC, 2015b). *S. enterica* serotype Enteritidis was the confirmed etiology of this outbreak and cases were localized in the Eastern U.S. with the highest case counts reported from Massachusetts (36) and New York (22). One illness occurred in Montana; however, this case had traveled to the Eastern U.S., where the exposure is suspected to have occurred. Illness onset occurred from September 30 to December 15, 2014, and of cases with information available, the hospitalization rate was 25.3%. The majority of cases were female (64%), and the median case age was 32, although ages ranged from one to 83 years of age. Epidemiological data indicated that bean sprouts were the likely food vehicle in this outbreak, and a common supplier was identified who destroyed the remaining product.

## Conclusion

In summary, a total of 85 multistate outbreaks that had a confirmed etiology and were associated with fresh produce occurred from 2010 to 2017 in the U.S. The outbreaks discussed in this review draw attention to factors that may contribute to the occurrence of multistate outbreaks. One such factor is that bacterial contamination can be propagated along the distribution chain, which may increase the scope of an outbreak and traceback difficulty. Specific examples include cross-contamination through the consolidation of fresh produce before distribution and in displays at the retail level. Another frequent contributor to multistate outbreaks in fresh produce was poor agricultural practices, especially in relation to melons. Sprouts were repeat offenders as food vehicles for various foodborne pathogens, and further regulation or more regular inspections may be needed to avoid future incidents. Imported fresh produce was also implicated as a food vehicle in several multistate outbreaks. The FDA has the authority to collect and analyze samples from imported goods as these products must meet FDA standards (FDA, 2018a). The FDA can also enact import alerts which enable the detention of imported goods without physical examination and require the importer to demonstrate that the product is in compliance. In the case of imported fresh produce, more frequent screening or import alerts may be necessary for these products to ensure that they are safe for consumption. The case-study included in this review underscores the risks associated with the use of fresh produce in multi-component food products. Surveillance of foodborne diseases and produce recall have proven critical to prevent and reduce the number of illnesses associated with outbreaks during the assessed timeframe.

As a result of a large multistate outbreak of *E. coli* O157:H7 more recently in 2018, the industry has initiated voluntary labeling of packaged Romaine lettuce with the growing region and harvest date per FDA recommendation (FDA, 2018b). The extension of this labeling practice to other fresh produce items would enable more effective traceback analysis and protect consumers in the case of future outbreaks. It is essential to pinpoint the causes and mechanisms by which contamination occurs in multistate outbreaks in order to prevent their occurrence. This task is difficult, as in some cases, the food vehicle is not confirmed; or a single source of contamination along the farm to fork continuum cannot be identified. In other cases, the root cause of the contamination is not isolated, which is critical for the development of future prevention measures.

## Author Contributions

CC worked on the literature review, data collection, data analysis, and wrote the manuscript. JS and CD analyzed the data, wrote the manuscript, and reviewed the manuscript.

## Conflict of Interest

The authors declare that the research was conducted in the absence of any commercial or financial relationships that could be construed as a potential conflict of interest.
